# Multiphysics Modeling of Plasmon-Enhanced All-Optical Helicity-Dependent Switching

**DOI:** 10.1021/acsphotonics.2c01815

**Published:** 2023-04-27

**Authors:** Feng Cheng, Chuangtang Wang, Yihao Xu, Wei Ma, Yongmin Liu

**Affiliations:** † Department of Electrical and Computer Engineering, Northeastern University, Boston, Massachusetts 02115, United States; ‡ Department of Mechanical and Industrial Engineering, Northeastern University, Boston, Massachusetts 02115, United States

**Keywords:** all-optical switching, plasmonics, multiphysics, Monte Carlo method, opto-magnetic, opto-thermal

## Abstract

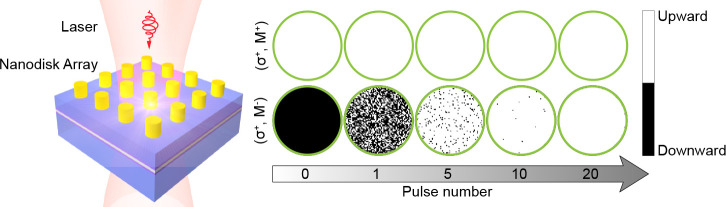

In this work, we propose a multiphysics approach to simulate all-optical helicity-dependent switching induced by the local hot spots of plasmonic nanostructures. Due to the plasmonic resonance of an array of gold nanodisks, strong electromagnetic fields are generated within the magnetic recording media underneath the gold nanodisks. We construct a multiphysics framework considering the opto-magnetic and opto-thermal effects, and then model the magnetization switching using the Monte Carlo method. Our approach bridges the gap between plasmonic nanostructure design and magnetization switching modeling, allowing for the simulation of helicity-dependent, nanoscale magnetization switching in the presence of localized surface plasmons.

## Introduction

The research of plasmonics has become an extremely fruitful subdiscipline in optics and photonics over the past two decades. By using rationally designed plasmonic structures, we can confine light into the dimension below the diffraction limit and enhance the local electric field intensity by orders of magnitude, which have led to major breakthroughs in several research fields, such as super-resolution imaging,^1,2^ lithography,^3^ biomedical sensing,^4,5^ and energy harvesting.^6,7^ In addition, interfacing plasmonics with magnetism has emerged as an exciting direction. Plasmonic nanodots/nanoholes^8−16^ and gratings^17−21^ were proposed to enhance magneto-optical responses, such as increasing magneto-optical Faraday/Kerr rotation angles. The use of plasmonic nanostructures has also been proposed for all-optical magnetization manipulation, in which magnetization can be directly controlled by light without any external magnetic fields.^22−31^ In this way, it is expected that data bits at the nanometer scale can be directly written with pulsed lasers, making all-optical, ultrafast, and high-density data storage possible. One special phenomenon of all-optical magnetization manipulation is termed all-optical helicity-dependent switching (AO-HDS). In AO-HDS, the magnetization can be deterministically and reversibly controlled by left-handed and right-handed circularly polarized laser pulses, opening a new door to store data bits directly with light.^32−34^ The AO-HDS phenomenon was initially found in ferrimagnetic materials composed of rare earth and transition metal elements.^35−50^ The existence of AO-HDS was later discovered in rare-earth-free ferromagnetic materials, such as Co/Pt multilayers and granular FePt films, which are of great importance for industrial-level applications of magnetic data storage.^50−59^

In previous work, researchers have designed different plasmonic nanostructures and investigated the polarization states of localized hot spots. However, direct modeling of magnetization switching induced by the localized chiral field is still lacking. One effective model that can analyze magnetization switching with external laser pulses is the Monte Carlo method, which has been utilized to simulate the heat-assistant magnetization reversal in ultrathin films,^60^ radiation-induced demagnetization,^61^ and all-optical switching.^62^ In the model of all-optical switching, the opto-thermal and opto-magnetic effects of lasers are considered. As a result, left-handed and right-handed circularly polarized lasers within a certain fluence range can deterministically switch the magnetization. In this work, we combine full-wave simulations with the Monte Carlo method to investigate magnetic switching in the presence of plasmon-induced local chiral fields that substantially enhance both opto-thermal and opto-magnetic effects. Our multiphysics modeling framework provides a very useful tool to investigate ultrafast and nanoscale AO-HDS in hybrid magneto-plasmonic systems.

## Method and Results

**Figure 1:**
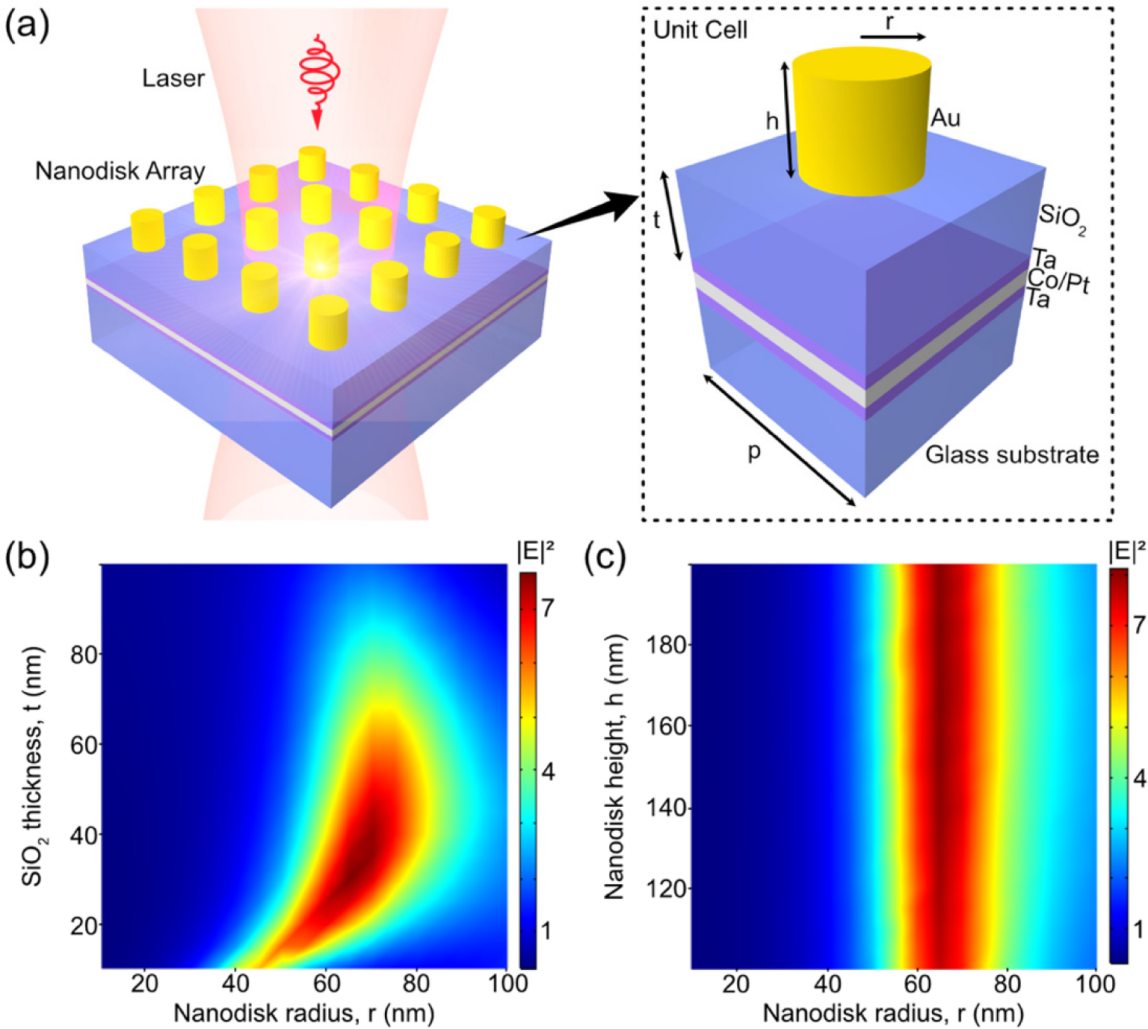
(a) Schematic view of the modeled system. A circularly polarized laser at the wavelength of 800 nm illuminates the sample and generates localized hot spots in the underlying Co/Pt layer. The ferromagnetic Co/Pt multilayer is sandwiched by the bottom and top capping layers of 3 nm Ta. The substrate is glass. Inset: unit cell of the structure. (b) Plot of the electric field intensity at the center of Co/Pt layer by sweeping the nanodisk radius and SiO_2_ thickness, when the nanodisk height is fixed at 100 nm. (c) Plot of the electric field intensity at the center of Co/Pt layer by sweeping the nanodisk radius and height, when the thickness of the SiO_2_ spacing layer is fixed at 30 nm.

To help better understand AO-HDS enhanced by plasmonic hot spots and further advance the field that interfaces nano plasmonics with ultrafast magnetism, here we propose a multiphysics model to simulate AO-HDS in the presence of localized electromagnetic fields generated by Au nanodisk arrays. The schematic of the system is shown in Figure 1a. We have designed the nanodisk arrays on top of a ferromagnetic thin film (i.e., 6 nm Co/Pt multilayer), which can generate strong local fields inside the Co/Pt layer upon laser illumination. Ta (3 nm in thickness) is used as the adhesive and capping layer underneath and above the Co/Pt multilayer, respectively. The inset of Figure 1a illustrates the unit cell of the nanodisk array. The radius and height of the nanodisk, and the thickness of the SiO_2_ spacing layer are denoted by *r*, *h*, and *t*, respectively. The periodicity of the unit cell is chosen as *p* = 600 nm. The design of the nanodisk geometry is performed by optimizing the geometric parameters and recording the electric field intensity at the center of the Co/Pt multilayer for each parameter combination. The incident laser is circularly polarized light at 800 nm wavelength, and the magnitude of electric field is 1 V m^–1^ in both the *x* and *y* directions. First, we have swept the nanodisk radius *r* from 10 to 100 nm, and the SiO_2_ thickness *t* from 10 to 100 nm, while the nanodisk height *h* is kept as 100 nm. The electric field intensity at the center of the Co/Pt layer is plotted in Figure 1b, which shows the maximum field intensity at *r* = 65 nm and *t* = 30 nm. Next, we have swept the nanodisk radius *r* from 10 to 100 nm, and the nanodisk height *h* from 100 to 200 nm, while keeping SiO_2_ thickness *t* at 30 nm. The electric field intensity recorded at the center of Co/Pt layer is depicted in Figure 1c. For a certain nanodisk radius, the electric field intensity does not change much when the height of nanodisk is varied. In order to get the optimized nanodisk height, we have swept it in a larger range from 50 to 350 nm with a finer resolution of 5 nm, with *r* = 65 nm and *t* = 30 nm. The maximum electric field intensity 8.23 V^2^ m^–2^ is achieved at *h* = 175 nm. From the above parameter sweeping procedures, we have found the optimized geometric parameters of the nanodisk, which are *r* = 65 nm, *t* = 30 nm, and *h* = 175 nm.

**Figure 2:**
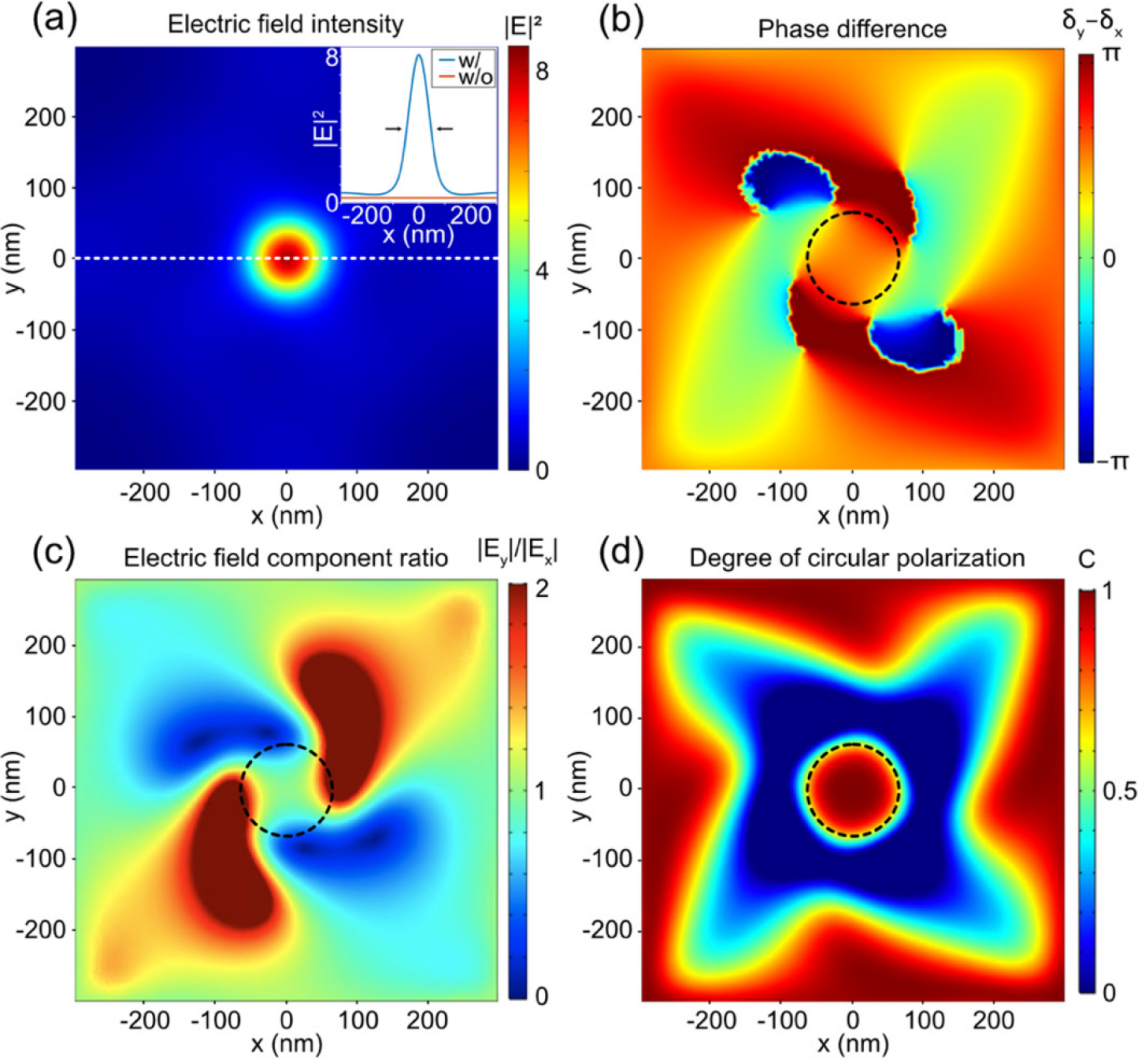
(a) Electric field intensity plotted in the middle of the Co/Pt layer with the optimized geometric parameters *r* = 65 nm, *t* = 30 nm, and *h* = 175 nm. The incident light is circularly polarized light with the electric field amplitude equal to 1 V m^–1^ in both the *x* and *y* directions. Inset: cross-section plot along the white dashed line for samples with and without the gold nanodisk array. (b–d) Distribution of the phase difference (δ_*y*_ – δ_*x*_), the electric field ratio |*E*_*y*_|/|*E*_*x*_|, and the degree of circular polarization in the middle of the Co/Pt layer.

Next, we investigate the hot spots generated in the magnetic thin film based on our optimized nanodisk array. Figure 2a plots the electric field intensity in the middle of Co/Pt layer, showing a maximum intensity of 8.23 V^2^ m^–2^ at the center. To quantify the plasmonic enhancement of the nanodisk array, we have simulated the electromagnetic field distribution for a bare Co/Pt sample. The cross-section plots of the electromagnetic fields of the samples with and without the nanodisk array are plotted as the inset of Figure 2a. From the intensity profile, we can clearly observe a hot spot for the sample with the nanodisk array. The full width of half-maximum of the hot spot is about 90 nm, and the maximum intensity at the center is enhanced by 30 times in comparison with the bare Co/Pt sample.

We have further investigated the polarization of the generated localized hot spot by analyzing the amplitude *E*_*x*_ (*E*_*y*_) and phase δ_*x*_ (δ_*y*_) of the *x*-component (*y*-component) of the electric field. Figure 2b shows the distribution of phase difference (δ_*y*_ – δ_*x*_) in the middle of the Co/Pt layer. The phase difference within the center region of the nanodisk is around π/2. Figure 2c presents the electric field intensity ratio |*E*_*y*_|/|*E*_*x*_| distribution in the middle of the Co/Pt layer. The field intensity ratio within the center region is about unity. The characteristics of the phase difference and intensity ratio indicate that the generated hot spot is circularly polarized. To study the polarization state of the hot spot in a quantitative manner, we have calculated the degree of circular polarization, which is defined as^25,63^
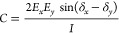
1Here *I* denotes the intensity of the electric field. A unity *C* corresponds to perfect circular polarization or chiral field. For fixed |*E*_*x*_| and |*E*_*y*_| and thus fixed intensity, *C* will increase when the polarization state changes from linearly polarized light to circularly polarized light. Figure 2d clearly shows that *C* is equal to unity in the center region of the Co/Pt layer, confirming the circular polarization of the generated hot spot. We can further introduce the figure of merit, which is defined as FOM = *IC*^2^.^25,63^ From the definition of FOM and the results shown in Figure 2a,d, it is apparent that the optimized gold nanodisk array can produce localized and enhanced circularly polarized light in the ferromagnetic Co/Pt layer.

After investigating the characteristics of the hot spot, we focus on the opto-magnetic and opto-thermal effects induced by the plasmonic hot spot. Here we use typical experimental parameters for laser repetition rate *f*, pulse duration τ, and beam diameter *d*, which are *f* = 200 kHz, τ = 200 fs, and *d* = 50 μm, respectively. The opto-magnetic effect of the laser pulse can be simulated by the inverse Faraday effect (IFE),^43,45,64^ that is,
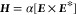
2Here ***E*** represents the electric field vector in the middle of the Co/Pt layer, and the magneto-optical susceptibility α is set to be 2.1 × 10^–11^ Am V^–2^ in our calculation.^64^ Next, we model the opto-thermal effect by the two-temperature model (TTM), which has been used widely to simulate the interaction between ultrafast laser and magnetic materials.^45,64,65^ The two temperatures correspond to the temperatures of the electron and phonon (lattice) systems, which are defined as *T*_e_ and *T*_p_, respectively. Mathematically, the TTM model can be written as^45^
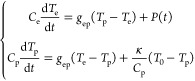
3In eq 3, the electron–phonon coupling coefficient is *g*_ep_ = 2.6 × 10^18^ W m^–3^ K^–1^, and the phonon heat capacity is *C*_p_ = 3 × 10^6^ J m^–3^ K^–1^.^64,65^ The electron heat capacity *C*_e_ is linearly proportional to the electron temperature *T*_e_, which is given by *C*_e_ = γ*T*_e_, with γ = 665 J m^–3^ K^–2^. The initial temperature is set as the room temperature *T*_0_ = 298 K, and we take κ = 1.8 × 10^24^ W^2^ m^–6^ k^–2^, which represents the energy dissipation to the environment. The laser irradiation *P*(*t*) is calculated from laser fluence *F*.

**Figure 3:**
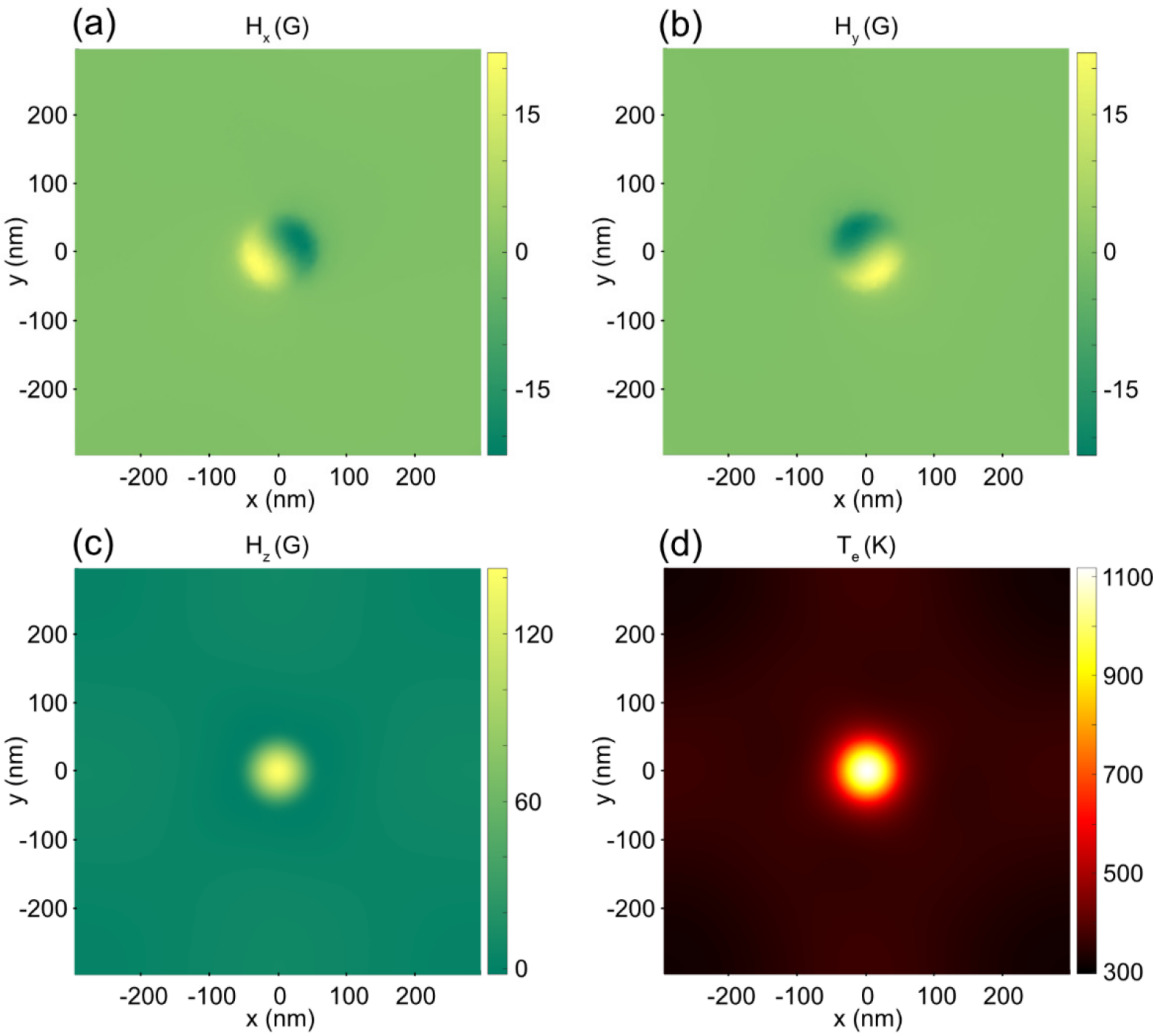
(a–c) Opto-magnetic effect of the local hot spot calculated from the inverse Faraday effect. The maximum opto-magnetic field components induced by one laser pulse along the *x*, *y*, and *z* directions are plotted in (a), (b), and (c) respectively. (d) Simulated distribution of maximum electron temperature *T*_e_ induced by one laser pulse based on the two-temperature model, which represents the opto-thermal effect of the hot spot. The input laser fluence is *F* = 7.6 μJ/cm^2^ for the calculations in (a)–(d).

To estimate the opto-magnetic effect generated by the localized hot spots, we have exported the real and imaginary parts of the electric fields in *x*, *y*, and *z* directions from the full-wave simulation by commercial software COMSOL Multiphysics and calculated the opto-magnetic field based on eq 2. Figure 3a,b shows the maximum *H*_*x*_ and *H*_*y*_ components of the induced opto-magnetic field by one laser pulse with right-handed circular polarization. The positive sign represents the direction of the opto-magnetic field along the +*x* or +*y* directions. At the center point, *H*_*x*_ and *H*_*y*_ are relatively small compared with the *H*_*z*_ component, as demonstrated in Figure 3c. Therefore, the magnetic field in the center primarily points along the out-of-plane direction, which triggers the magnetization switching in the Co/Pt layer with perpendicular magnetic anisotropy. Subsequently, we have exported the intensity profile from the COMSOL full-wave simulation and calculated the opto-thermal effect based on eq 3. The spatial distribution of maximum electron temperature *T*_e_ induced by one laser pulse is shown in Figure 3d. With a laser fluence 7.6 μJ/cm^2^, *T*_e_ reaches up to about 1100 K. Since the Curie temperature of CoPt is *T*_c_ = 550 K,^65^ such a laser irradiation is able to induce magnetization switching, as we will show in the following. Additionally, the induced electron temperature has a Gaussian-like intensity distribution, which is similar to the localized electromagnetic field distribution. Recently, it has been shown that surface lattice resonance can be utilized to control the distribution of electric fields and thereby regulate the efficacy of ultrafast demagnetization and all-optical switching.^28,30,66^ Although we focus on localized surface plasmon resonance in the present work, our multiphysics model can be readily applied to investigate the influence of surface lattice resonance on ultrafast magnetism at the nanoscale.

**Figure 4:**
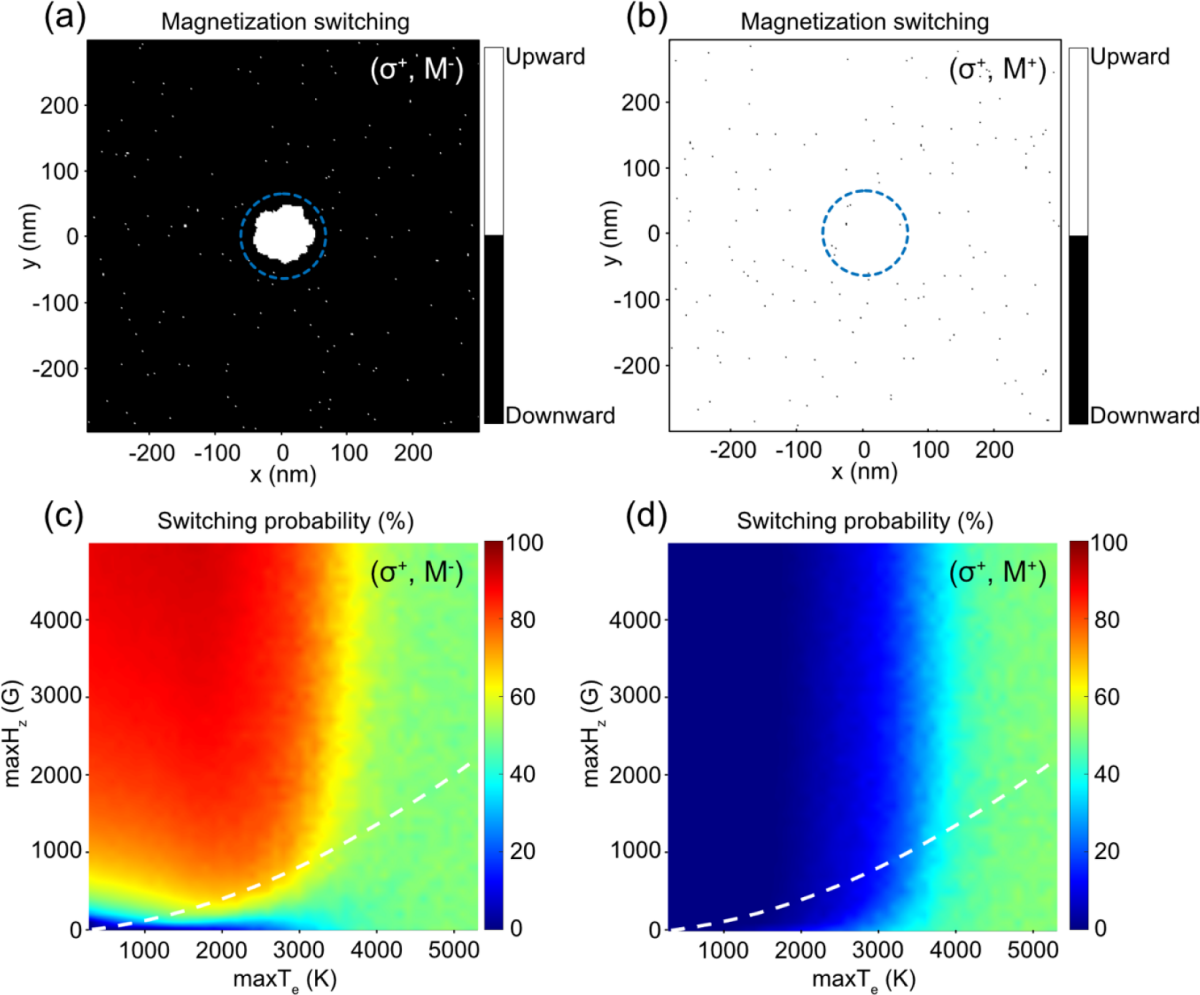
(a, b) Examples of magnetization switching simulated by the Monte Carlo method, when a single σ^+^ laser pulse illuminates on initially (a) M^–^ and (b) M^+^ magnetization. The input laser fluence is *F* = 7.6 μJ/cm^2^. The blue dashed circle indicates the designed nanodisk. (c, d) Phase map of the switching probability as a function of the maximum *T*_e_ and maximum *H*_*z*_ for (c) (σ^+^, M^–^) and (d) (σ^+^, M^+^). The white dashed line represents the parameter pair of (max*T*_e_, max*H*_*z*_) that can be generated by a single laser pulse. The switching probability is calculated by averaging the results from 100 Monte Carlo trials.

After the calculation of opto-magnetic and opto-thermal effects of the laser, we simulate the magnetization switching by the Monte Carlo method.^60−62,67,68^ More information can be found in the Supporting Information. Figure 4a shows the magnetization switching for configuration (σ^+^, M^–^), which means the input light is right-handed circularly polarized and the initial magnetization points downward. After a single right-circularly polarized pulse with laser fluence *F* = 7.6 μJ/cm^2^, a large portion of the spins within the center region is flipped due to the localized chiral hot spot. As a comparison, the magnetization switching for configuration (σ^+^, M^+^), that is, right-circularly polarized pulse incident on upward magnetization, at the same laser fluence, is shown in Figure 4b. Almost no spins are flipped in this case.

In order to quantitatively describe such magnetization switching at the nanometer scale, we have calculated the switching probability, which is defined as the portion of the switched magnetization within the central area indicated by the blue dashed circle in Figure 4a,b. The radius of the circle is chosen as *R* = 65 nm, which is the radius of the designed nanodisk. The switching probability as a function of the electron temperature and the *z*-component of the opto-magnetic field for the (σ^+^, M^–^) configuration is shown in Figure 4c. The switching probability is calculated by sweeping all configurations of the max*T*_e_ and max*H*_*z*_ parameters, with max*T*_e_ ranging from 300 to 5300 K and max*H*_*z*_ ranging from 0 to 5000 G. max*T*_e_ and max*H*_*z*_ denote the maximum electron temperature and the maximum *z*-component of opto-magnetic field used for Monte Carlo simulation, respectively, and the percentage value is calculated by averaging the results from 100 Monte Carlo trials. From Figure 4c, it can be observed that when max*T*_e_ is less than about 3000 K, the switching probability will gradually increase as magnetic field max*H*_*z*_ increases. When max*H*_*z*_ is larger than about 3000 G and max*T*_e_ is less than about 2000 K, the switching probability can achieve over 90%. Another observation is that when max*T*_e_ is larger than about 3500 K, the switching probability is about 50%. It is reasonable since the opto-thermal effect is so strong in this case, which dominates the process and leads to thermal demagnetization. The switching probability for the (σ^+^, M^+^) configuration is shown in Figure 4d. Compared with the (σ^+^, M^–^) configuration, the switching probability is almost 0%, regardless of the value of max*H*_*z*_ when max*T*_e_ is less than about 3000 K. When max*T*_e_ is larger than about 3500 K, the switching probability of about 50% can also be observed, which is caused by thermal demagnetization.

The white dashed lines in Figure 4c,d show a parameter pair of (max*T*_e_, max*H*_*z*_) that can be generated by a single laser pulse (with fluence increases from 0 to 75 μJ/cm^2^). max*T*_e_ and max*H*_*z*_ are calculated by TTM and IFE correspondingly. Even though effective magnetization switching cannot be achieved with a single laser pulse, the switching probability difference between (σ^+^, M^–^) and (σ^+^, M^+^) indicates that effective magnetization switching can be realized with accumulative laser pulses.^69^ In order to investigate such accumulative switching with multiple pulses, we have calculated the cumulative probability as a function of the laser pulse number based on the equation below:^69^
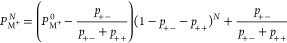
4Here *P*_M^+^_^*N*^ denotes the cumulative probability in the case of initially upward magnetization after *N* pulses, whereas *P*_M^+^_^0^ represents the percentage of upward magnetization before the laser exposure. We consider that the initial magnetization points downward, so *P*_M^+^_^0^ = 0. And *p*_+–_ and *p*_++_ denote the switching probability for configuration (σ^+^, M^–^) and (σ^+^, M^+^), respectively.

**Figure 5:**
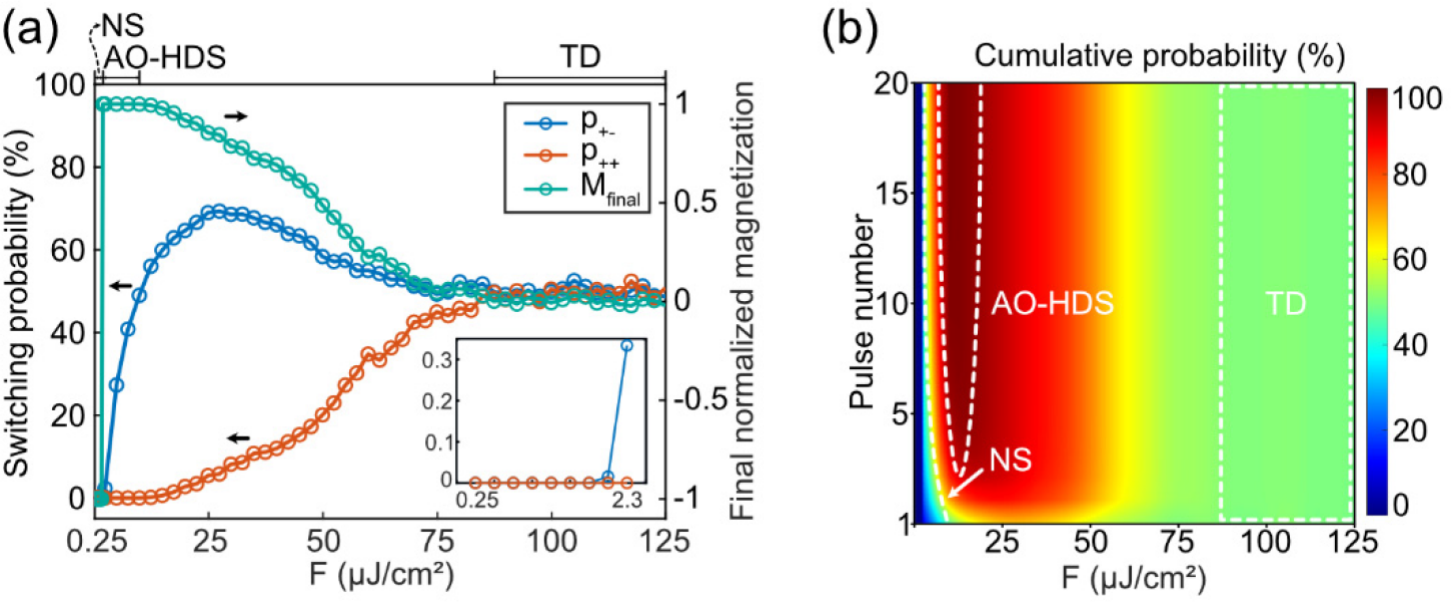
(a) Switching probability (*p*_+–_ and *p*_++_) and final normalized magnetization (M_final_) as a function of the laser fluence. The inset shows the zoomed in plot of the “NS” zone. (b) Cumulative probability as a function of the laser fluence and number of laser pulses. NS: nonswitching; AO-HDS: all-optical helicity-dependent switching; TD: thermal demagnetization.

We have simulated the switching probability along the white dashed lines for a single laser pulse in Figure 4c,d, and the results are plotted in Figure 5a. For the (σ^+^, M^–^) configuration, the switching probability is 0% when the laser fluence is very small. When increasing the laser fluence, the switching probability increases and reaches a maximum percentage of 69.4%. When the laser fluence further increases, the switching probability reduces and eventually drops to around 50% due to thermal demagnetization. On the other hand, the switching probability for (σ^+^, M^+^) shows 0% when the laser fluence is too small. The switching probability gradually increases when laser fluence is increased and becomes stabilized at around 50% when thermal demagnetization occurs.

After multiple laser pulses the two configurations reach the equilibrium state, the final normalized magnetization M_final_ is only related to the switching probability of two configurations (σ^+^, M^–^) and (σ^+^, M^+^) and can be calculated by the following equation:^56^
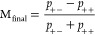
5The final normalized magnetization as a function of laser fluence is also plotted in Figure 5a. From the switching probability curves, we can recognize three fluence zones that produce three distinct results, namely, “NS” zone for nonswitching, “AO-HDS” zone for all-optical helicity-dependent switching, and “TD” zone for thermal demagnetization. When the laser fluence falls in the “NS” zone, the switching probabilities for both configurations are 0%. Therefore, no magnetization switching happens, and the final normalized magnetization remains to be −1 since the initial magnetization points downward. A very large switching probability (approaching 100%) takes place when the laser fluence falls in the “AO-HDS” zone, where the switching probability for (σ^+^, M^–^) is nonzero but it is still zero for (σ^+^, M^+^). Consequently, the final normalized magnetization becomes 1. Finally, when laser fluence falls in the “TD” zone, thermal demagnetization happens, and the final normalized magnetization becomes 0.

The cumulative probability *P*_M^+^_^*N*^ as a function of the laser fluence and number of laser pulses is shown in Figure 5b. Similar to the observation from the M_final_ curve in Figure 5a, when the laser fluence falls in the “NS” zone, the magnetization will not be switched no matter how many laser pulses are applied. When the laser fluence falls in the “AO-HDS” zone, 100% switching probability can be achieved after accumulative laser pulses. When the laser fluence is further increased into the “TD” zone, the switching probability becomes about 50%.

**Figure 6:**
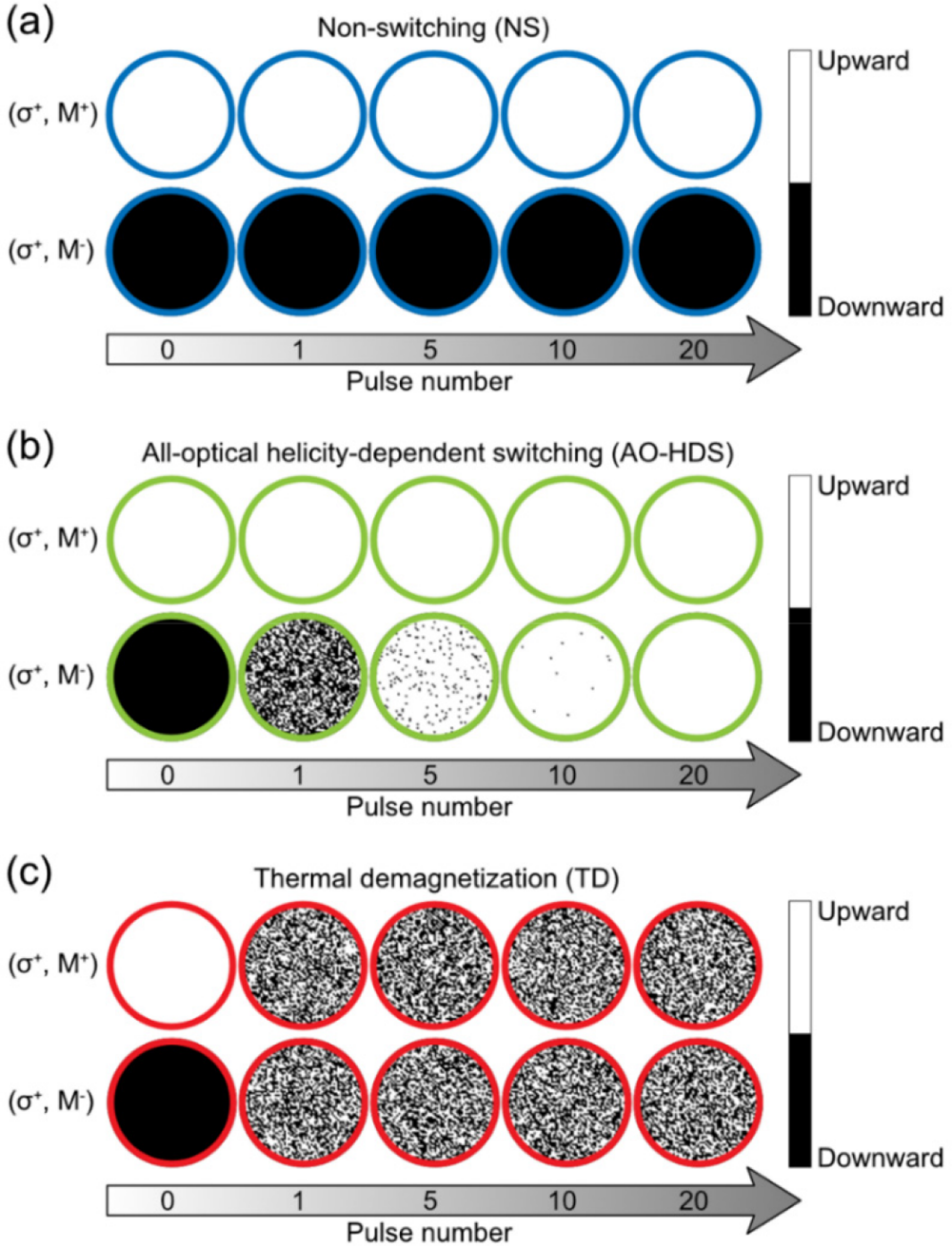
Evolution of magnetization subject to multiple laser pulses for (σ^+^, M^–^) and (σ^+^, M^+^). NS (marked by blue), AO-HDS (marked by green), and TD (marked by red) are shown in (a)–(c), respectively. The radius of the circle is 65 nm.

To directly visualize the characteristics of “NS”, “AO-HDS”, and “TD” zones, we present the magnetization within the central circular region (radius *R* = 65 nm) after the illumination of multiple laser pulses. As shown in Figure 6a, when the laser fluence falls in the “NS” zone, nonswitching is observed in both cases. Interestingly, as plotted in Figure 6b, when the laser fluence falls in the “AO-HDS” zone, we can see that magnetization switching gradually establishes with increasing pulses for (σ^+^, M^–^) while no magnetization switching happens for (σ^+^, M^+^). This result clearly shows the accumulative helicity-dependent switching behavior. However, when the laser fluence falls in the “TD” zone, thermal demagnetization is observed for both (σ^+^, M^–^) and (σ^+^, M^+^), as shown in Figure 6c.

## Conclusion

In summary, in this paper we have designed plasmonic Au nanodisk arrays to achieve local hot spots with well-defined circular polarization states in the magnetic recording media. A multiphysics model is introduced to simulate the plasmon-enhanced all-optical helicity-dependent switching. We have systematically studied the opto-magnetic and opto-thermal effects of the laser pulse by the inverse Faraday effect and two-temperature model, respectively. The resulting opto-magnetic and opto-thermal effects are then utilized to simulate the magnetization switching with the Monte Carlo method. Our multiphysics model is a very useful tool to study ultrafast, nanoscale magnetic switching induced by plasmonic nanostructures. It would help us to design future low-power and high-density all-optical magnetic data storage devices.

## Supporting Information Available

Procedures of the multiphysics modeling and details of the full-wave simulation and Monte Carlo simulation (PDF)
