# Supplementary material for: Multiphysics Modeling of Plasmon-Enhanced All-Optical Helicity-Dependent Switching
Source: ACS Photonics. 2023 Apr 27;10(5):1259–67. doi: 10.1021/acsphotonics.2c01815 (PMC10621044; doi:10.1021/acsphotonics.2c01815)
Supplement: Supplementary file 1 — ph2c01815_si_001.pdf [file ph2c01815_si_001.pdf]

Supporting Information

Multiphysics Modeling of Plasmon-Enhanced All-Optical Helicity-Dependent Switching

Feng Cheng[1], Chuangtang Wang[1], Yihao Xu[2], Wei Ma[2], and Yongmin Liu[1,2,*]

[1]Department of Electrical and Computer Engineering, Northeastern University, Boston, Massachusetts 02115, USA
[2]Department of Mechanical and Industrial Engineering, Northeastern University, Boston, Massachusetts 02115, USA
[*] Email: y.liu@northeastern.edu

Simulation of magnetization switching with a multiphysics approach

We propose a multiphysics approach to simulate the magnetization switching induced by plasmonic hot spots. The multiphysics approach, as shown in Figure S1, is composed of two parts: (1) "Full-Wave Simulation" and (2) "Monte Carlo Simulation."

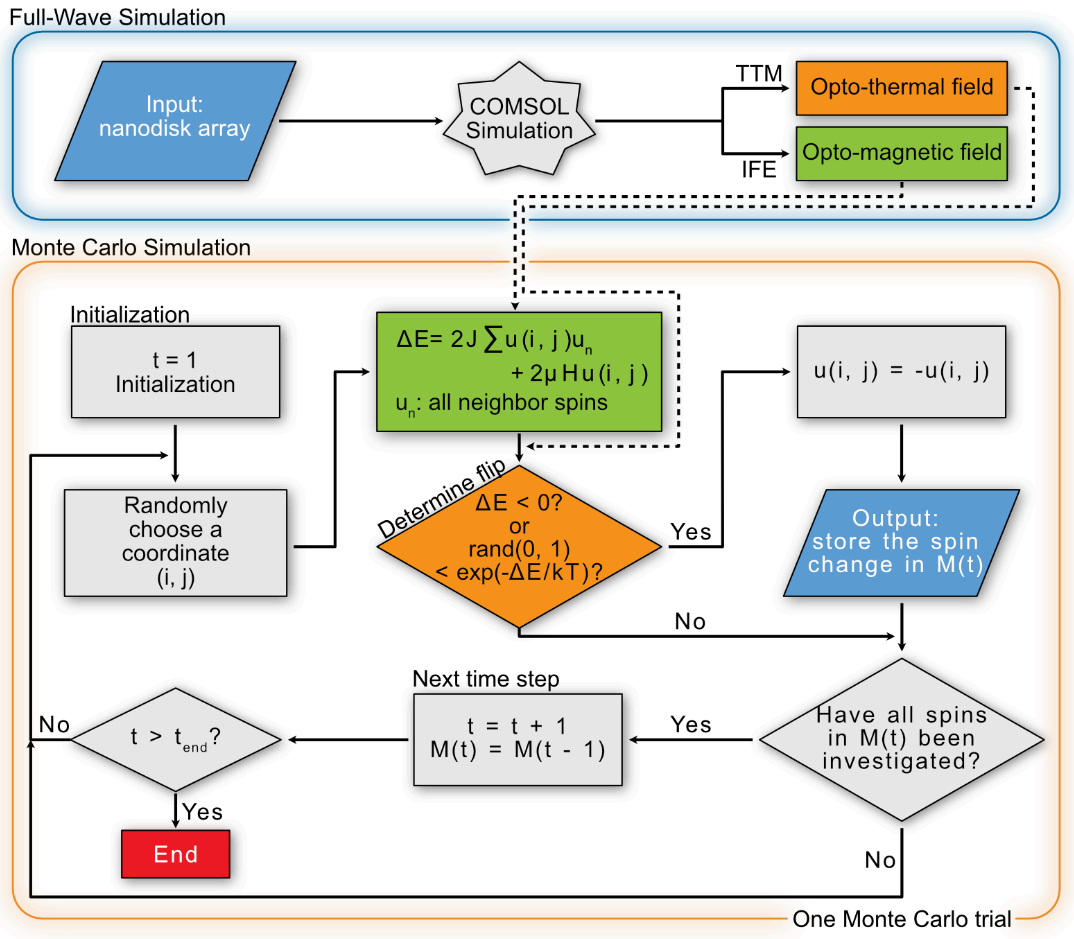

Figure S1. Flow chart of the multiphysics approach to simulate the magnetization switching induced by plasmonic hot spots. The multiphysics approach is composed of two parts: "Full-Wave Simulation" and "Monte Carlo Simulation."

In the full-wave simulation, we first simulate the local electromagnetic field distributions in our system, that is, a Co/Pt multilayer integrated with an array of Au nanodisks, by commercial electromagnetic solver COMSOL Multiphysics. We have utilized periodic boundary conditions to model one unit cell of the nanodisk arrays, and used circularly polarized light as excitation. The Co/Pt multilayer is modeled as an effective medium with permittivity retrieved from ellipsometry measurement. The permittivities of Au, Ta, and SiO_2 are also taken from experimental data. Figure S2 summarizes the permittivities of materials used in the simulations.

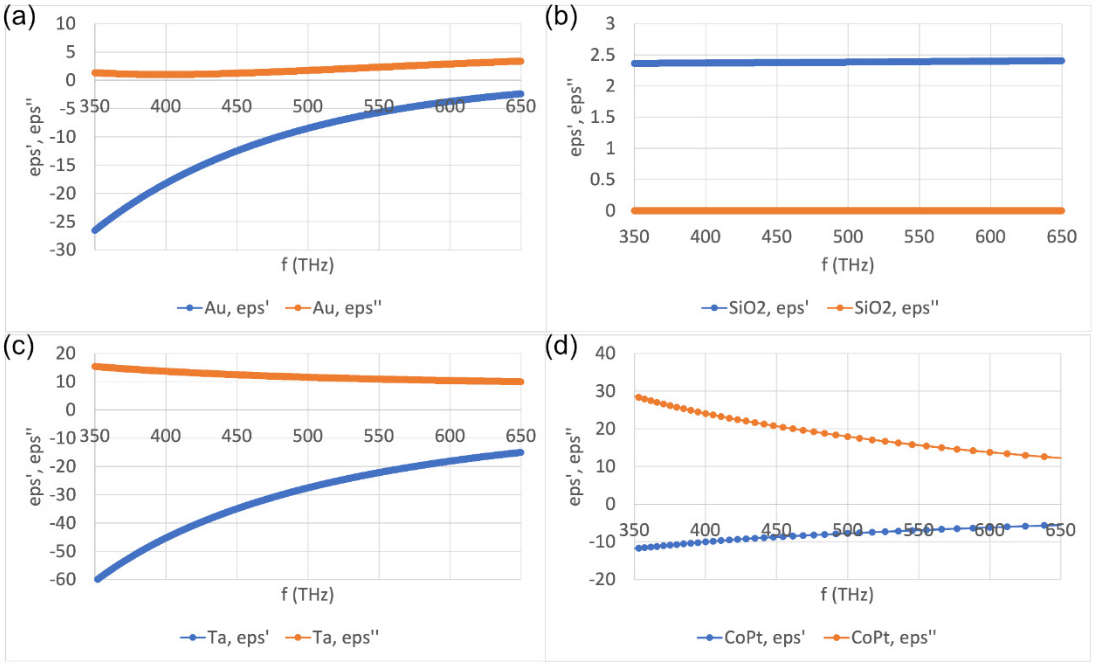

Figure S2. Relative permittivities of materials used in simulations. (a) Au, (b) SiO_2, (c) Ta, and (d) Co/Pt.

We then export the electromagnetic field components (E_x, E_y, E_z, H_x, H_y, H_z) within the plane cut through the middle of the Co/Pt layer from COMSOL and calculate the two-fold effects from the localized hot spots. As discussed in the main manuscript, the opto-magnetic field is calculated with inversed Faraday effect (IFE), and the opto-thermal field is calculated with the two-temperature model (TTM).

Next, we model the magnetization switching induced by plasmonic hot spots via Monte Carlo simulation. As marked by the dashed lines in Figure S1, we import the opto-magnetic and opto-thermal fields into the Monte Carlo algorithm. The Monte Carlo simulation begins with initialization of the ferromagnetic lattice. Here the simulation domain has the same size as the unit cell of the nanodisk array (600 nm × 600 nm) with periodic boundary conditions. We have chosen the spin size as 2 nm × 2 nm so that the lattice can be considered as a 301 × 301 matrix. Before the simulation of each Monte Carlo trial, the whole domain is initialized so that all spins point to either upward or downward direction.

After initialization, we apply the metropolis algorithm [S1] to simulate the magnetization switching. As illustrated in the flow chart, the algorithm is processed as follows:

First, we define the total energy of the spin system with Hamiltonian

$$E = -J \sum_n u(i,j) u_n - \mu H u(i,j) \tag{S1}$$

The first term in Equation S1 represents the local coupling energy of the spin system, n denotes the neighboring spins for the spin at coordinate (i, j), and J is the strength of the spin coupling. For a system with $J > 0$, the spins tend to align along the same direction for a lower total energy. The second term represents the Zeeman energy, where H is the opto-magnetic field generated from the plasmonic hot spots. For a system with $\mu > 0$, the spins tend to stay aligned with opto-magnetic field H for a lower energy. We have used $J = 0.02$ eV and $\mu = 4.2\times10^{-4}$ eV/G in the simulation.

Then, a spin at coordinate (i, j) is randomly selected and flipped. The flip of spin $u(i, j)$ means that the new energy needs to be calculated with $-u(i, j)$ based on Equation S1. Therefore, the change of energy is given by:

$$\Delta E = 2J \sum_n u(i,j)u_n + 2\mu H u(i,j) \tag{S2}$$

After the calculation of the energy change, the algorithm determines the flip based on the following flip probability (see reference [S1] and [S2]):

$$P = min\left(1, exp\left(\frac{-\Delta E}{k_B T}\right)\right) \tag{S3}$$

where k_B is the Boltzmann constant, and T denotes the opto-thermal field generated from the plasmonic hot spots. When $\Delta E < 0$, the switching probability $P = 1$, meaning that the spin will be flipped. This is physically reasonable since the new state has a lower total energy. When $\Delta E > 0$, the spin will flip with a probability P larger than a generated random number between 0 to 1. The larger the probability P is, the more likely the spin will flip.

After investigating the flip condition for one spin $u(i,j)$, we store the spin change in the magnetization matrix $M(t)$. Then the algorithm checks if all spins in the 301 × 301 matrix have been investigated. If not, the algorithm randomly chooses another spin and check the flip condition.

When all spins have been investigated, we apply the algorithm to the next time frame with $t = t + 1$, and save the current magnetization as the initial condition for the next time frame $M(t) = M(t-1)$. The algorithm then goes through the previous process to flip spins in the matrix. The algorithm ends when the time is larger than the end time. The output will be the final magnetization matrix.

Reference

[S1]I. Petrila and V. Manta, "Metropolis Monte Carlo analysis of all-optical switching," *Computer Physics Communications*, vol. 185, no. 11, pp. 2874–2878, Nov. 2014, doi: 10.1016/j.cpc.2014.07.008.

[S2]W. R. Deskins, G. Brown, S. H. Thompson, and P. A. Rikvold, "Kinetic Monte Carlo simulations of a model for heat-assisted magnetization reversal in ultrathin films," *Phys. Rev. B*, vol. 84, no. 9, p. 094431, Sep. 2011, doi: 10.1103/PhysRevB.84.094431.
